# Clot composition of embolic strokes of undetermined source: a feasibility study

**DOI:** 10.1186/s12883-020-01969-w

**Published:** 2020-10-21

**Authors:** Amre Nouh, Tapan Mehta, Mohamed Hussain, Xianyuan Song, Martin Ollenschleger

**Affiliations:** 1 Department of Neurology, Hartford Hospital, University of Connecticut, 80 Seymour Street, Hartford, CT 06115 USA; 2 Department of Neurology, University of Minnesota, Minneapolis, MN USA; 3 Department of Neuroradiology, Hartford Hospital, University of Connecticut, Hartford, CT USA; 4 Department of Pathology, Hartford Hospital, University of Connecticut, Hartford, CT USA

**Keywords:** Embolic stroke of undetermined source, Mechanical Thrombectomy, Large vessel occlusion, Cryptogenic stroke, Clot

## Abstract

**Background:**

A number of emerging studies have evaluated clot composition in acute ischemic stroke. Studies of clot composition of embolic strokes of undetermined strokes are lacking.

**Objectives:**

We sought to analyze the RBC to platelet ratios in clots and correlated our findings with stroke etiology.

**Methods:**

This was a prospective study analyzing clots retrieved by mechanical thrombectomy in acute ischemic stroke patients at our institution. All clots were stained and scanned at 200x magnification by using a Scanscope XT digital scanner (Apergio, Vista, California). Image-J software (National Institutes of Health, Bethesda, Maryland) was used for semi quantitative analysis of percentage RBC’s and platelets. Unpaired t-test was used to compare means of RBC to Platelet ratios. Correlation of RBC to Platelet ratios with stroke etiology was performed.

**Results:**

A total of 33 clots from 33 patients were analyzed. Stroke etiology was undetermined in 6 patients, cardioembolic in 14, large vessel atherosclerosis (LVA) in 9, and carotid dissection in 4. The mean RBC to platelet ratio was 0.78:1 (+/− 0.65) in cardioembolic clots, 1.73:1 (+/− 2.38) in LVA and 1.4:1(+/− 0.70) in carotid dissections. Although patients with undetermined etiology had a similar clot composition to cardioembolic stroke (0.36:1+/− 0.33), (*p* = 0.19), it differed significantly from LVA and dissections respectively (*p* = 0.037, *p* = 0.01).

**Conclusion:**

In our study, a low RBC to Platelet ratio was found among patients with embolic strokes of undetermined source, however shared similar characteristics with cardioembolic thrombi. Ongoing collection and analysis is needed to confirm these findings and its significance in evaluating stroke etiology.

## Background

As the treatment of acute ischemic stroke in patients with large vessel occlusion (LVO) has established the efficacy of mechanical thrombectomy, the number of clot retrievals have increased and will continue to rise as the window for endovascular intervention has been expanded to 24 h from symptom onset in eligible patients [1, 2]. In turn, an increase in the number of emerging studies analyzing retrieved clots for histopathological makeup and the clinical significance they harbor has been observed. Most frequently, studies have reported the relationship between clot histology; angiographic outcomes post thrombectomy, imaging characteristics during acute stroke and ultimately stroke etiology. A large meta-analysis that reviewed over 25 studies and several hundred patients found no association between histopathological characteristics of thrombi and stroke etiology or angiographic outcomes [3]. However correlation with etiology has varied so has techniques and quantitative analysis methods; a limitation noted by the authors of the metanalysis and observed abroad these studies.

As up to a third of all ischemic strokes remain cryptogenic or of unknown etiology, evaluating emboli from patients with LVO’s may shed some light on the potential underlying embolic mechanism [4]. In particular, patients with embolic strokes of undetermined source (ESUS); a relatively new classification highlighting patients with ischemic strokes of truly undetermined source are a subset of patients were studying clot pathology would be extremely valuable. ESUS refers to non-lacunar infarct (subcortical infarct ≤1.5 cm on CT or ≤ 2.0 cm on MRI) in the absence the following: extra-cranial or intracranial atherosclerosis ≥50% luminal stenosis in the artery supplying the ischemic region, major cardioembolic sources [permanent or paroxysmal atrial fibrillation (AF), sustained atrial flutter, intracardiac thrombus, prosthetic cardiac valve, atrial myxoma or other cardiac tumors, mitral stenosis, myocardial infarction within the past 4 weeks, left ventricular (LV) ejection fraction < 30%, valvular vegetation’s or infective endocarditis], and no other specific cause of stroke (e.g., dissection, arteritis, migraine/vasospasm, drug misuse) [5].

A growing body of literature has proposed several similarities between cardioembolic stroke mechanisms and ESUS. Several biomarkers of atrial dysfunction associated with embolic stroke independent of atrial fibrillation have been reported including elevations in serum N-terminal BNP [6–8], p-wave dispersion of ECG [9], PR interval prolongation [10], left atrial fibrosis [11, 12] and left atrial appendage morphology [13]. In addition, genetic characteristics in conjunction with infarction location in patients with cryptogenic vs. cardioembolic, arterial and lacunar strokes have predicted probable cardioembolic stroke in 58% of cryptogenic strokes based on similar RNA expression profiles [14]. Furthermore, with stroke recurrence as high as 29% in patients with ESUS [15], additional investigations that may lead to a possible underlying etiology are extremely valuable to direct future appropriate secondary prevention.

In our study, we sought to investigate the histopathology of patients with ESUS compared with patients with known etiologies. We postulated that similar to the characteristics shared between cardioembolism and ESUS, the histopathology would also be similar and differ from other causes of LVO.

## Methods

A prospective two-phase study evaluating all patients with acute ischemic stroke treatment undergoing mechanical thrombectomy with an established LVO was designed. After institutional IRB approval, data and specimen collection in January of 2016 had started. All patients with an intact clot specimen appropriate for preparation and staining with complete data after chart review were included. Patients with partial or destroyed specimens and those with missing data were excluded. Stroke etiology during the patient’s hospitalization was determined by two independent fellowship trained physician based on chart evaluation and defined as cardioembolic, large vessel disease, and embolic of undetermined source based on the aforementioned ESUS criteria. Data collection included patients’ demographics, comorbidities, clinical presentation, imaging findings, laboratory parameters, stroke severity and time of presentation, treatment methods, location of LVO, recanalization scored by TICI scores (thrombolysis in cerebral infarction), imaging findings, and clinical outcomes. Only patients with complete datasets and intact clot specimens were included in the prospective collection process.

**Fig. 1:**
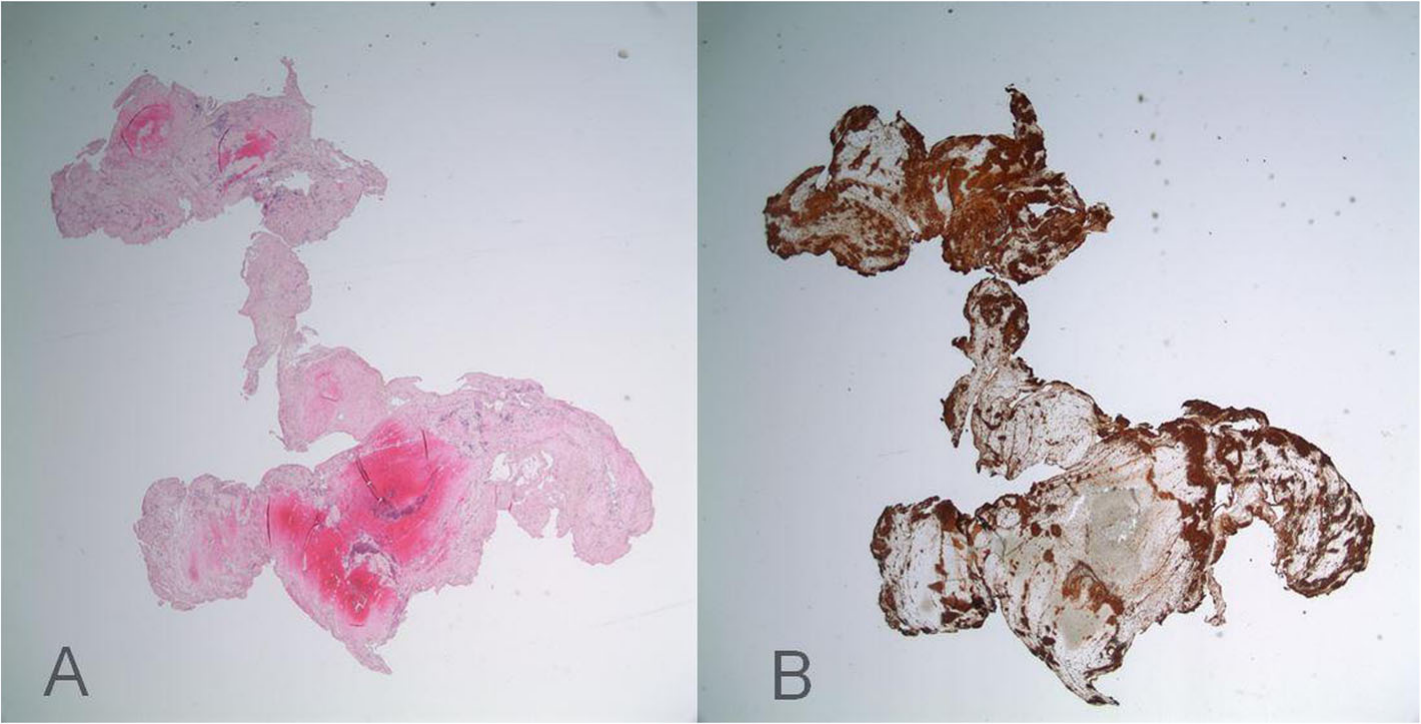
Histopathologic stained specimen of a clot. Stained slides were scanned at 200x magnification by using a Scanscope XT digital scanner (Apergio, Vista, California). Panel A with Hematoxylin-eosin to identify red blood cells (RBC’s) staining pink. Panel B CD61 (LifeSpan Biosciences, Seattle, Washington) antibodies for platelet glycoprotein IIIa staining brown

Intact specimens successfully retrieved by mechanical thrombectomy were processed and prepped by a blinded pathologist to the patient’s etiology, clinical characteristics and outcomes. Retrieved clot material was fixed and cut at 4-m thickness and all clots were stained with hematoxylin-eosin to identify red blood cells (RBC’s), and antibodies for platelet glycoprotein IIIa with CD61 (LifeSpan Biosciences, Seattle, Washington) for platelets. Several slices per specimen were evaluated and were scanned at 200x magnification by using a Scanscope XT digital scanner (Apergio, Vista, California). For quantitative analysis, Image-J software (National Institutes of Health, Bethesda, Maryland) was used to evaluate the percentage RBC’s and platelets in all specimens (Fig. 1). For each specimen, a mean percentage RBC and Platelet (fibrin) was calculated (based on the area of staining for markers of each cell type) after evaluating all slides per specimen. Out of 33 clot samples, 26 samples had single slide, 6 samples had two slides and one clot had four slides. All percentages were referenced to a platelet ratio of 1 in compared to proportion RBCs. Shapiro-Wilk test was applied to test normality of RBC to Platelet ratio values distribution in each stroke etiology subgroup. Due to the non-normal distribution of RBC to Platelet ratios in the large vessel atherosclerosis group, initially all the means were compared using independent sample Kruskal-Wallis Test. Subsequently comparison of RBC to Platelet ratio means between ESUS Vs Cardioembolic groups and ESUS Vs Dissection groups was performed using independent sample T test (equal variances assumed). The RBC to Platelet ratio means for ESUS Vs large vessel atherosclerosis group were compared using Mann-Whitney U test. Patient demographic and co-morbidities were compared for all four groups. One way ANOVA test was applied to compare the difference in the mean age. Fischer’s Exact test was applied to compare the categorical variables described in Table 2. Statistical significance was set at 0.05. An interim analysis of 18 clots from 18 patients was completed in May of 2017 during our studies first phase to establish feasibility and consistent methodology and has been previously reported [16]. Data is available upon reasonable request to corresponding author.

## Results

After reviewing inclusion and exclusion criteria, a total of 33 specimens from 33 patients were collected and analyzed. Stroke etiology was found to be ESUS in 18.2% (*n* = 6), cardioembolic in 42.5% (*n* = 14), Large-vessel atherosclerosis (LVA) in 27.25% (*n* = 9), and carotid dissection in 12% (*n* = 4). Shapiro-Wilk test was applied to test the normality of distribution of the RBC to Platelet ratio values in each subgroup. All except LVA group were noted to have normal distribution. The mean values of RBC to Platelet ratio among four groups were noted to have statistically significant difference on Kruskal-Wallis Test (ESUS 0.36, SD 0.33; Cardioembolic 0.78, SD 0.65; LVA 1.73, SD 2.38; Carotid Dissection 1.44, SD 0.70; *P* Value – 0.037). On individual group comparisons, the mean RBC to Platelet ratio of ESUS group was significantly different from LVA (Mann-Whiney U Test, *p* = 0.026) and Carotid dissection (Independent sample T Test, *p* = 0.01); whereas, it was not significantly different from the cardioembolic group (Independent sample T test, *p* = 0.19) Table 1.

**Table 1:** Mean platelet to RBC ratio of clot by etiology of stroke

Etiology	*N* = 33	Mean Platelet/RBC Ratio	SD +/−	*P* Value
Cardioembolic (afib)	14	1:0.789	0.66	0.15^#^
ESUS^a^	6	1:0.361	0.34	–
Large vessel Atherosclerosis	9	1:1.727	2.39	0.03^@^
Dissection	4	1:1.442	0.70	0.01^#^

^a^ESUS- embolic strokes of undetermined source

^#^ *p* value using independent Sample T Test

^@^ *p* value using Mann-Whitney U test

Although patient characteristics and comorbidities may differ between stroke etiology subgroups, in our study only atrial fibrillation was observed to be more frequent among cardioembolic stroke, as expected. The absence of significant differences among patient groups may be due in part to the small study population. Table 2 outlines patient characteristics in respect to comorbidities and stroke subtypes.

**Table 2:** Comorbidity distribution based on etiology of stroke

	ESUS [6]	Cardioembolic [14]	Large Vessel Atherosclerosis [10]	Cervicocephalic Dissection [3]	*P* value
Age in Years [Mean, (Standard deviation)]	69.17 (17.8)	74.71 (6.87)	62.60 (15.62)	49.33 (12.42)	0.02
n (% of total cases of the same etiology)					
Clot location					0.67
Anterior circulation (M1, M2 or intracranial internal carotid artery)	4 (66.7)	11 (78.6)	9 (90)	2 (66.7)	
Basilar artery	2 (33.3)	3 (21.4)	1 (10)	1 (33.3)	
Gender (Male)	2 (33.3)	7 (50)	6 (60)	3 (100)	0.28
Race					0.42
Caucasian	4 (66.7)	16 (92.9)	9 (90.0)	2 (66.7)	
African American	0 (0)	0 (0)	0 (0)	0 (0)	
Hispanic	1 (16.7)	0 (0)	1 (10)	1 (33.3)	
Other	1 (16.7)	1 (7.1)	0 (0)	0 (0)	
Coronary artery disease	1 (16.7)	3 (21.4)	1 (10)	0 (0)	0.75
Congestive Heart Failure	1 (16.7)	4 (28.6)	1 (10)	0 (0)	0.54
Atrial Fibrillation	0 (0)	11 (78.6)	0 (0)	1 (33.3)	< 0.001
Hypertension	3 (50)	12 (85.7)	7 (70)	1 (33.3)	0.2
Hyperlipidemia	4 (66.7)	8 (57.1)	6 (60)	2 (66.7)	0.97
Diabetes	1 (16.7)	3 (21.4)	1 (10)	0 (0)	0.76
Smoking	1 (16.7)	3 (21.4)	2 (20)	1 (33.3)	0.95
Alcohol	1 (16.7)	3 (21.4)	2 (20)	1 (33.3)	0.95
Pre hospitalization anti-platelet use	1 (16.7)	3 (21.4)	3 (9.1)	0 (0)	0.72
Pre hospitalization anticoagulation	0 (0)	4 (28.6)	0 (0)	1 (33.3)	0.13
Pre hospitalization statin use	3 (50)	5 (35.7)	2 (20)	1 (33.3)	0.66

## Discussion

The importance of histopathological analysis of the thrombi retrieved during treatment of acute ischemic stroke due to LVO has been investigated [17, 18]. Our study suggests that clot analysis may be helpful to identify stroke etiology in line of previously published data [19, 20]. We found that an RBC to Platelet ratio among patients with LVA or dissection is significantly higher compared to ESUS. On the other hand, clots with underlying etiology as cardioembolic thrombi had no difference in RBC to platelet ratio when compared with ESUS. Our study attempts to address several important issues for future the clot analysis protocols despite smaller sample size [21].

One of the biggest confounders for such an investigation is appropriate sample collection and it’s processing. The concept of analyzing clots retrieved during LVO treatment is not novel, however, the devices and thrombectomy methods has evolved significantly in the past [17, 22, 23]. The newer generation stent retriever devices along with supplementary methods including suction or proximal balloon occlusion have shown better complete recanalization rates surrogating for more representative samples retrieved during thrombectomy [24]. There is a considerable variation in thrombectomy techniques based on provider preference and angioarchitecture at the time of intervention [23].

The majority of the samples collected in our study were retrieved using stent retriever device with or without the use of aspiration. However, there are several unmeasured confounders. For an example, the stent retriever itself could act as a thrombogenic material depending on the brand, metal content of the device, and duration of deployment ultimately affecting the composition of the captured clot. The method for analysis of the clot sample is an important issue and there is a significant variability. Considering that a single section might not be representative of the entire clot composition, we attempted to include more than one section of the clot sample if feasible in the analysis to improve the accuracy of clot composition analysis [25]. Although the use of antibody based highly specific stains could be more accurate, the feasibility and increase the cost of the analysis makes this challenging. Similarly, the clot analysis could be done using sophisticated devices including electron microscope, the large-scale implementation of such protocols could be difficult.

In our pre specified clot analysis, we only attempted to analyze its impact on understanding the etiology of the stroke. Our study was aimed toward the feasibility of clot analysis process and the findings are limited due to small sample size. However, others have also examined the clot composition and its radiographic appearance, the effect of tPA on clot composition, and histological indications for thrombolytic therapy resistance [26–28].

## Conclusion

Our study suggests the potential association of clot composition with the stroke etiology using a small sample size. It also raises the importance of easily accessible, cost effective and reliable method of clot analysis which can be standardized and generate consistent data and larger registries for future analysis. As the field advances, the utility of clot composition will increase and hopefully help establish etiology for patients with embolic strokes of undetermined source.

## Data Availability

All data is available upon request to the corresponding author.

## Abbreviations

LVA: Large vessel atherosclerosis
RBC: Red blood cell
LVO: Large vessel occlusion
ESUS: Embolic strokes of undetermined source
AF: Atrial fibrillation
TICI: Thrombolysis in cerebral infarction
